# Precision Treatment in ACS–Role of Assessing Fibrinolysis

**DOI:** 10.3390/jcm10050929

**Published:** 2021-03-01

**Authors:** Ying X. Gue, Young-Hoon Jeong, Mohamed Farag, Nikolaos Spinthakis, Diana A. Gorog

**Affiliations:** 1 Liverpool Centre for Cardiovascular Science, University of Liverpool and Liverpool Heart & Chest Hospital, Liverpool L14 3PE, UK; y.gue@liverpool.ac.uk; 2 Department of Life and Medical Sciences, University of Hertfordshire, Hatfield AL10 9AB, UK; mohamedfarag@nhs.net (M.F.); nspinthakis@nhs.net (N.S.); 3 Department of Internal Medicine, Gyeongsang National University School of Medicine and Cardiovascular Center, Gyeongsang National University Changwon Hospital, Changwon 51472, Korea; goodoctor@naver.com; 4 National Heart and Lung Institute, Imperial College, London SW3 6LY, UK

**Keywords:** endogenous fibrinolysis, precision medicine, acute coronary syndrome

## Abstract

Despite advancements in pharmacotherapy and interventional strategies, patients with acute coronary syndrome (ACS) remain at risk of recurrent thrombotic events. In addition to an enhanced tendency to thrombus formation, impairment in the ability to naturally dissolve or lyse a developing thrombus, namely impaired endogenous fibrinolysis, is responsible for a major part of this residual risk regardless of optimal antiplatelet medication. Global assessment of endogenous fibrinolysis, including a point-of-care assay, can identify patients with ACS at persistent high cardiovascular risk and might play an important role in allowing the personalisation of potent antithrombotic therapy to enhance fibrinolytic status, providing precision treatment of ACS to improve long-term outcome.

## 1. Background

The mainstay management of acute coronary syndrome (ACS) involves the use of antithrombotic therapy to reduce the risk of adverse ischaemic events [1]. International guidelines for antithrombotic treatment for ACS patients recommend administration of dual antiplatelet therapy (DAPT), comprising of aspirin together with a P2Y_12_ inhibitor [2,3]. However, despite advancements in pharmacotherapy and interventional strategies, approximately 10% of ACS patients remain at risk of major adverse cardiovascular events (MACE) after an ACS, a risk mainly driven by thrombotic events [4,5]. This was initially attributed to reduced antiplatelet effect with high on-treatment platelet reactivity (HTPR), potentially due to variability of individual responsiveness to antiplatelet agents [6]. However, although there appeared to be a correlation between observed HTPR and adverse events [7], randomised studies utilising platelet function test (PFT) to guide management have not significantly improved clinical outcomes [8,9]. The TRILOGY ACS (Targeted Platelet Inhibition to Clarify the Optimal Strategy to Medically Manage Acute Coronary Syndromes) PFT (VerifyNow P2Y12 assay) sub-study showed that even with a more potent P2Y_12_ inhibitor like prasugrel, which resulted in lower platelet reactivity when compared to clopidogrel (median 64; interquartile range (IQR), 33–128 P2Y12 reaction unit (PRU) vs. 200; IQR, 141–260 PRU; *p* < 0.001), there was no significant difference in clinical outcomes (cardiovascular death, myocardial infarction and stroke) at 30 months (17.2% vs. 18.9%, *p* = 0.29). This shows that the residual ischemic risk in these high-risk patients cannot be simply explained by HTPR [5].

Haemostasis is a complex sequence of biochemical responses to injury, which facilitates formation of a blood clot and reparation of the damaged endothelium. The maintenance of the equilibrium between coagulation and fibrinolysis is critical to health, as any imbalance may lead to either abnormal bleeding, resulting in haemorrhage, or thrombosis resulting in ischemia. Attempts to manage and prevent thrombotic events have mainly focused on preventing the formation of a thrombus—namely with the use of antiplatelet and/or anticoagulant agents. Whilst treatment with DAPT addresses the enhanced platelet reactivity in ACS, ongoing activation of the coagulation cascade and impaired endogenous fibrinolysis appears to be unaffected by the current DAPT strategy [10].

In addition to the activation of coagulation, the inflammatory pathway has been identified to contribute to the residual ischemic risk in patients with atherosclerotic disease [11]. The CANTOS (Canakinumab Anti-Inflammatory Thrombosis Outcome Study) included stabilized myocardial infarction (MI) patients with persistent high inflammatory risk (high-sensitivity C-reactive protein ≥2 mg/L) treated with canakinumab, a monoclonal antibody targeting interleukin-1β, demonstrated a significant reduction in MACE with canakinumab compared with patients received placebo (hazard ratio (HR) 0.85, 95% confidence interval (CI), 0.74 to 0.98; *p* = 0.021) [12]. Indeed, both the inflammation and coagulation pathways contribute to the ischemic risk and there has been increasing evidence of cross-over between the two pathways (i.e., inflammation leads to activation of coagulation, and coagulation considerably impacts on the inflammatory activity) [13,14]. Furthermore, and apart from the increased tendency to thrombus formation (enhanced platelet reactivity), impairment in the ability to naturally dissolve or lyse a developing thrombus, namely impaired endogenous fibrinolysis, is responsible for a major part of the remaining residual risk in patients with ACS on optimal antiplatelet medication [15].

## 2. Assessment of Fibrinolysis

### 2.1. Assessment of Individual Enzymes and Proteins That Mediate Fibrinolysis

The role of impaired endogenous fibrinolysis in ACS is well recognized [16,17]. Several biomarkers, namely factors regulating fibrinolysis (such as tissue plasminogen activator (t-PA), plasminogen activator inhibitor 1 (PAI-1), and lipoprotein A (Lp(a))) have been investigated in various studies. A higher level of t-PA correlated with an increased risk of MACE post-ACS [18]. Moreover, patients with ACS exhibited higher t-PA levels when compared to patients with stable angina [19]. PAI-1 levels independently predicted in-hospital and one-year mortality in patients with ACS [20], and their increased levels were associated with higher rates of all-cause mortality and MI [21]. However, these results were not observed in other studies [22,23]. Similarly with Lp(a), previous studies have shown weak correlations [22] whereas more recent studies have not shown any association of Lp(a) level and mortality [24]. These conflicting results show the complexity of assessing the fibrinolytic activity using individual enzymes and proteins [16] and hence are of limited utility in advancing personalised medicine.

### 2.2. Global Assessment of Lysis

#### 2.2.1. Turbidimetric Clot Lysis Assay

As the structure of the fibrin network regulates the mechanical stability of the thrombus and its resistance to fibrinolysis, the assessment of fibrin clot structure can provide information about thromboembolic risk and susceptibility to lysis [25]. Turbidimetric lysis analysis uses citrated plasma to which agonists added—a calcium/thrombin buffer—to induce clot formation, and t-PA added to induce fibrinolysis. Fibrin clot lysis is the measure of time to achieve lysis of the clot and typically the speed at which 50% lysis occurs, as well as maximal absorbance, are measured. Assessment of fibrinolysis with plasma clot lysis has been shown to independently predict recurrent MI and cardiovascular death in all-comers following ACS [26] and also specifically in ACS patients with diabetes [27]. However, the use of plasma rather than whole blood, means that the impact of cellular constituents of blood on lysis is not assessed. Furthermore, the addition of agonists and t-PA to induce thrombosis and fibrinolysis means that in fact, this technique detects the plasma’s ability to respond to external fibrinolytic treatment, as opposed to measuring endogenous, native fibrinolysis. Additionally, the test is cumbersome and requires dedicated and experienced laboratory staff trained to perform this reliably, making it impracticable in the clinical setting.

#### 2.2.2. Thromboelastography and Thromboelastometry

Thromboelastography (TEG^®^: Haemonetics, UK) and rotational thromboelastometry (ROTEM^®^: Pentapharm GmbH, Munich, Germany) are point-of-care, global tests of coagulation status, simultaneously assessing clot development, stabilization, and dissolution/lysis, both based on the same principles. These techniques utilize a pin suspended by a torsion wire into a cylinder to measure the physical properties of a clot. As blood clot formation occurs around the pin, fibrin strands form between the cylindrical cup and pin. The rotation of the cylindrical cup is transmitted to the pin, whose displacement is then picked up by the torsion wire. This is analysed and presented in graphical form by the instrument to allow analysis of different stages of coagulation and fibrinolysis [28]. The technique is able to measure clot formation time and rate, maximum amplitude or clot strength and clot lysis time. TEG can be performed using native blood sample or with modification such as with the addition of urokinase [29] which can improve detection of fibrinolysis but this has only been performed in patients with sepsis [30] and may therefore be of lesser clinical relevance in the ACS setting. More recently, the ClotPro^®^ (enicor GmbH, Munich, Germany) which utilizes viscoelastic principles but allows the use of manufacturer provided reagents to test different aspects of the coagulation pathway could bring about more clinical uses [31].

TEG is well-established in detecting hyperfibrinolysis, namely bleeding risk, but its success is limited in the assessment of hypofibrinolysis or thrombosis risk [32,33]. Another TEG shortcoming is the employment of the low-flow, static-type conditions, which resemble more venous rather than arterial thrombosis. This is less reflective of the physiological response to high shear thrombosis that occurs in ACS.

#### 2.2.3. Global Thrombosis Test

The global thrombosis test (GTT; Thromboquest Ltd., London, UK) is an automated, point-of-care test that simultaneously assesses platelet reactivity, thrombosis, and fibrinolytic activity, from a native whole blood sample subjected to high shear [34]. Blood inserted into the instrument’s cartridge passes through a plastic conical tube where narrow gaps create high shear stress that mimics flow within a narrowed vessel, activating platelets and inducing occlusive thrombus formation. Thrombus formation gradually reduces and finally occludes flow in the cartridge. Arrest of flow, as detected by an optical sensor, is expressed as occlusion time (OT). Blood flow resumes following spontaneous fibrinolysis, and the time taken to do so is expressed as lysis time (LT). The GTT therefore provides a comprehensive evaluation of thrombosis and lysis under high shear stress.

The main advantage of GTT is that it is an easy-to-use point-of-care test that mimics arterial thrombosis and can assess platelet reactivity, thrombus stability and endogenous fibrinolysis, providing an overall global assessment. The use of native, non-anticoagulated whole blood allows the measurement of the effects of thrombin generation in platelet aggregation without depletion of calcium (as opposed to citrated blood which is commonly required in other tests). Secondly, the presence of high shear as the key initiator of platelet activation is analogous to the physiological mechanism of platelet activation within a stenosed artery. Lastly, the assessment of spontaneous lysis through the measurement of LT is comparable to the physiological recanalization of an occluded artery. The main disadvantage is the requirement of immediate analysis of the native blood sample, which means samples cannot be stored for subsequent analysis.

## 3. Impaired Endogenous Fibrinolysis and Cardiovascular Risk

Several avenues of observational studies indicate that impaired endogenous fibrinolysis is associated with adverse outcomes. Of note, unfavourable fibrin clot properties that render fibrin clots more resistant to lysis are correlated with prothrombotic characteristics such as hypertension, diabetes, hyperlipidemia and smoking [35]. There has been increasing evidence that impaired endogenous fibrinolysis is a strong predictor of residual thrombotic risk in patients with ACS. In the RISK PPCI study, involving nearly 500 patients with ST-segment elevation MI, impaired endogenous fibrinolysis as measured using GTT, was detected in 14% of patients on admission and was strongly related to recurrent MACE (HR 9.1, 95% CI 4.28–15.03, *p* = 0.001), driven by cardiovascular death and MI [10]. In a different cohort of 300 ACS patients, impaired endogenous fibrinolysis was associated with an increased risk of MACE at 12 months (HR 2.52, 95% CI 1.34–4.71, *p* = 0.004) [36]. These studies provide a link between impaired endogenous fibrinolysis and residual cardiovascular risk. Furthermore, ACS patients with impaired fibrinolysis were shown to produce in vitro clots that, under electron microscopy, comprised of more highly branched, denser fibrin meshwork than patients with effective lysis potential [37]. In a sub-study of >4000 patients in the PLATO (Study of Platelet Inhibition and Patient Outcomes) trial, assessment of plasma clot lysis using a turbidimetric assay revealed that impaired fibrin clot lysis was an independent predictor of adverse outcome in ACS [26]. After adjusting for established cardiovascular risk factors, each 50% increase in lysis time was associated with cardiovascular death/spontaneous MI (HR 1.17, 95% CI 1.05–1.31; *p* < 0.01) and cardiovascular death alone (HR 1.36, 95% CI 1.17–1.59; *p* < 0.001). Similarly, in the sub-study involving diabetic patients, after adjusting for cardiovascular risk factors, each 50% increase in lysis time was associated with increased risk of cardiovascular death or MI (HR 1.21; 95% CI 1.02–1.44; *p* = 0.026) and cardiovascular death alone (HR 1.38; 95% CI 1.08–1.76; *p* = 0.01) [27].

These studies point towards the potential of using the assessment of endogenous fibrinolysis as a biomarker to identify patients who may be at increased risk of future adverse thrombotic events. This could allow the use of more potent antithrombotic pharmacotherapy in these patients to reduce the thrombotic risk.

## 4. ACS Risk Stratification Based on Fibrinolysis Assessment

The role of risk stratification in ACS patients is not new. There are various ways of stratifying ACS patients including the use of risk scores to determine patients who may be suitable for outpatient investigations and treatment [38], early aggressive treatment [39] and duration of antithrombotic therapy [40]. Many of these risk scores make use of clinical characteristics such as age and co-morbidities, which are commonly overlapping in both thrombotic and bleeding risks, therefore have limited use in accurately risk stratifying patients into high- versus low-risk in either bleeding or thrombotic risk. Biomarkers such as cardiac troponins assess short-term cardiovascular risk, but are not helpful beyond the acute hospitalisation and do not influence the choice of pharmacotherapy to reduce the thrombotic risk or personalise treatment.

The assessment of endogenous fibrinolysis may allow for personalised and targeted therapy whereby patients who are identified as having impaired endogenous fibrinolysis (higher thrombotic risk compared to bleeding risk) could be offered more potent pharmacotherapy to enhance fibrinolysis to reduce ischaemic cardiovascular events, while accepting a potential increased bleeding risk, in favour of an overall favourable risk-to-benefit ratio in high-risk individuals. Similarly, perhaps those who are identified as having enhanced endogenous fibrinolysis could be offered less potent pharmacotherapy to reduce the potential risk for bleeding. This strategy may allow ACS patients to gain the net benefit and reduce the overall risk of future complications.

## 5. Pharmacological Modulation of Endogenous Fibrinolysis

Unlike the enhanced platelet reactivity in ACS patients which improves in response to antiplatelet therapy, impaired fibrinolysis in ACS patients appears to be unaffected by DAPT [10,26].

A previous study assessing the effect of different P2Y_12_ inhibitors (oral clopidogrel, ticagrelor and intravenous cangrelor) on endogenous fibrinolysis in patients with coronary artery disease found that although all drugs reduced platelet reactivity, only cangrelor enhanced fibrinolysis, potentially through thrombus destabilization [41]. However, in 2 different studies exploring the effect of oral anticoagulants (OAC) on endogenous fibrinolysis in patients with atrial fibrillation, all OACs showed a signal towards enhancing endogenous fibrinolysis although only apixaban achieved a significant improvement in lysis [42,43]. It remains to be investigated whether the combination of antiplatelets and oral anticoagulants further enhances endogenous fibrinolysis.

## 6. The Effect of Anticoagulation on Cardiovascular Risk

There is evidence for activation of the coagulation cascade in patients presenting with ACS with elevation of biomarkers of coagulation activation including increased levels of d-dimer [44] and fibrinogen [45]. The link between higher d-dimer levels and the risk of subsequent stent thrombosis indirectly supports the theory of an enhanced coagulation cascade driving residual risk [16]. Furthermore, there is evidence that use of oral anticoagulation in patients with established cardiovascular disease reduces cardiovascular risk. Addition of warfarin to antiplatelet therapy in patients with ACS achieved a reduction in thrombotic events and death (16.7% vs. 20%, 95% CI 0.69–0.95, *p* = 0.03) but at the expense of increased bleeding (4.9% vs. 1.7%, *p* < 0.001) [46]. Furthermore, in the ATLAS ACS-TIMI 51 (Anti-Xa Therapy to Lower Cardiovascular Events in Addition to Standard Therapy in Subjects with Acute Coronary Syndrome–Thrombolysis in Myocardial Infarction 51) study, the addition of rivaroxaban 2.5 mg b.i.d. to DAPT in patients with a recent ACS resulted in a reduction in cardiovascular mortality (2.7% vs. 4.1%, *p* = 0.002), but also at the expense of major bleeding (2.1% vs. 0.6%, *p* < 0.001) and intracranial haemorrhage (0.6% vs. 0.2%, *p* = 0.009) [47]. In the COMPASS (Cardiovascular Outcomes for People Using Anticoagulation Strategies) study, among patients with stable atherosclerotic vascular disease, those assigned to rivaroxaban (2.5 mg twice daily) plus aspirin had fewer adverse cardiovascular outcomes, comprising of cardiovascular death, MI and stroke, than those assigned to aspirin alone, but with an increase in bleeding risk [48].

It is possible that the reduction in ischaemic events with OAC is achieved through enhancing endogenous fibrinolysis. Therefore, whilst OAC is clearly not be suitable for all patients, in order to achieve maximum net clinical benefit, ACS risk stratification to identify patients at higher ischemic risk is pertinent. The ongoing VaLiDate-R (can very low dose Rivaroxaban (VLDR) in addition to dual antiplatelet therapy improve thrombotic status in acute coronary syndrome?) study [49] (ClinicalTrials.gov Identifier: NCT03775746, EudraCT: 2018-003299-11) aims to assess whether the addition of vascular dose rivaroxaban can favourably enhance endogenous fibrinolysis in patients with recent ACS.

## 7. Precision Treatment in ACS Based on Fibrinolytic Status

**Figure 1:**
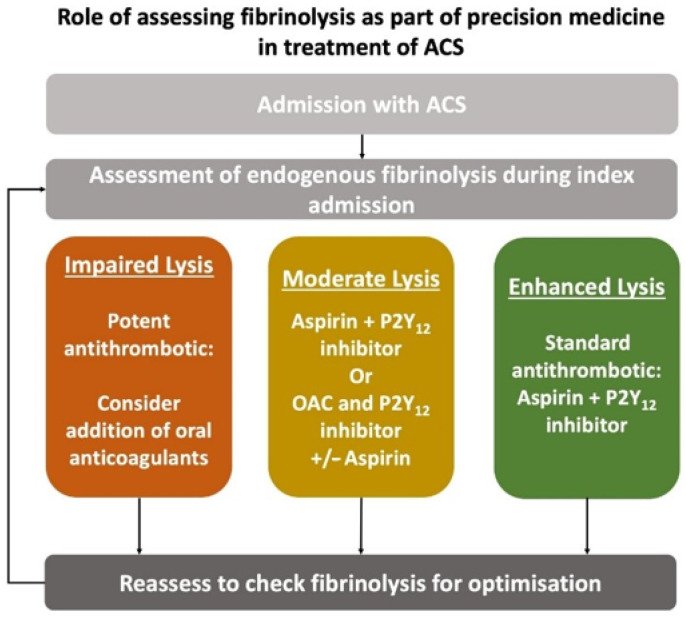
Proposed algorithm for assessing endogenous fibrinolysis in patients presenting with acute coronary syndrome (ACS). OAC: Oral anticoagulants.

Apart from the potential as a risk stratification tool in ACS, the assessment of endogenous fibrinolysis could enable personalised “precision” medicine in these patients (Figure 1). Patients at high ischemic risk, as determined by markedly impaired fibrinolysis, may derive the greatest benefit from use of more potent antithrombotic therapy than is conventionally employed, for example by addition of OAC to antiplatelet therapy. On the other hand, patients at low ischemic risk could avoid the increased risk of bleeding complications and may benefit from de-escalation of the intensity of antithrombotic therapy. Being able to risk stratify patients would empower clinicians to decide on the optimal pharmacotherapy to achieve the net clinical benefit, by balancing the risks of thrombosis and bleeding in a particular individual. Point-of-care testing of fibrinolytic status could facilitate the assessment of future ischaemic risk and allow timely monitoring and personalisation of pharmacotherapy.

## 8. Conclusions

The assessment of endogenous fibrinolysis can identify patients with ACS at high thrombotic risk and might play an important role in allowing the personalisation of pharmacotherapy to enhance fibrinolytic status, providing precision treatment of ACS to improve long-term outcome.

## Data Availability

Not applicable.
